# Metabolite Changes in an Estuarine Annelid Following Sublethal Exposure to a Mixture of Zinc and Boscalid

**DOI:** 10.3390/metabo9100229

**Published:** 2019-10-15

**Authors:** Georgia M. Sinclair, Allyson L. O’Brien, Michael Keough, David P. de Souza, Saravanan Dayalan, Komal Kanojia, Konstantinos Kouremenos, Dedreia L. Tull, Rhys A. Coleman, Oliver A.H. Jones, Sara M. Long

**Affiliations:** 1 School of Biosciences, The University of Melbourne, Royal Parade, Parkville Victoria 3010, Australia; s3762567@student.rmit.edu.au (G.M.S.); allyson@unimelb.edu.au (A.L.O.); mjkeough@unimelb.edu.au (M.K.); sara.long@rmit.edu.au (S.M.L.); 2 Centre for Aquatic Pollution Identification and Management (CAPIM), School of Biosciences, The University of Melbourne, Royal Parade, Parkville Victoria 3010, Australia; 3 Metabolomics Australia, Bio21 Molecular Science and Biotechnology Institute, 30 Flemington Road, Parkville, Victoria 3052, Australia; desouzad@unimelb.edu.au (D.P.d.S.); saravanan.dayalan@gmail.com (S.D.); komal.kanojia@unimelb.edu.au (K.K.); KKouremenos@trajanscimed.com (K.K.); dedreia@unimelb.edu.au (D.L.T.); 4 Melbourne Water Corporation, 990 La Trobe Street, Docklands, Victoria 3008, Australia; Rhys.Coleman@melbournewater.com.au; 5 Australian Centre for Research on Separation Science (ACROSS), School of Science, RMIT, University, GPO Box 2476, Melbourne, Victoria 3001, Australia

**Keywords:** annelid, biomonitoring, ecotoxicology, fungicide, metal, mixtures

## Abstract

Environmental pollutants such as heavy metals and fungicides pose a serious threat to waterways worldwide. Toxicological assessment of such contaminants is usually conducted using single compound exposures, as it is challenging to understand the effect of mixtures on biota using standard ecotoxicological methods; whereas complex chemical mixtures are more probable in ecosystems. This study exposed *Simplisetia aequisetis* (an estuarine annelid) to sublethal concentrations of a metal (zinc) and a fungicide (boscalid), both singly and as a mixture, for two weeks. Metabolomic analysis via gas and liquid chromatography-mass spectrometry was used to measure the stress response(s) of the organism following exposure. A total of 75 metabolites, including compounds contributing to the tricarboxylic acid cycle, the urea cycle, and a number of other pathways, were identified and quantified. The multiplatform approach identified distinct metabolomic responses to each compound that differed depending on whether the substance was presented singly or as a mixture, indicating a possible antagonistic effect. The study demonstrates that metabolomics is able to elucidate the effects and mode of action of contaminants and can identify possible outcomes faster than standard ecotoxicological endpoints, such as growth and reproduction. Metabolomics therefore has a possible future role in biomonitoring and ecosystem health assessments.

## 1. Introduction

Identifying the potential toxicity associated with complex mixtures of chemicals continues to be a major challenge for ecotoxicological assessments due to the underlying complexity of such exposures. Understanding the effect caused by mixtures of chemicals to an individual species, population, or community is, however, important to understand in order to conduct robust environmental risk assessments and make effective management decisions [1,2]. The difficulty is due, at least in part, to the fact that the toxicity of chemical mixtures is a result of unpredictable interactions between individual components and other chemicals and biomolecules within the study system. For example, exposure to a substance may induce biochemical changes that cause higher or lower sensitivity to subsequent exposures [1]. Synergistic and antagonistic interactions can also cause changes in overall toxicity compared to single-contaminant exposures [3]. It is essential, therefore, to develop new tools to understand and predict mixture effects to strengthen environmental risk assessments [4]. 

Metabolomics (the study of small biological molecules in a system) is increasingly being used as part of a weight-of-evidence approach for environmental assessments for two main reasons. The first is the large amount of information that can be extracted, detecting particular pathways and highlighting the modes of action a chemical can have within an organism. The second is the ability of metabolomics to detect sublethal biochemical changes in an organism quickly and before irreversible effects occur [5,6,7]. Development of molecular (e.g., genomic, proteomic or metabolomic) markers of exposure can, potentially, provide an overview of contaminant exposure on systems before other more commonly used ecotoxicological endpoints, such as growth, reproduction, or mortality [6]. For example, Jeppe et al. exposed *Chironomous tepperi*, a non-biting midge, to bifenthrin (a pyrethroid insecticide) over a five-day period [5]. They did not detect changes in growth or behavior but observed a change in molecular gene expression profiles. Similarly, Sinclair et al. identified metabolomic changes in *Simplisetia aequisetis* (an estuarine annelid) following exposure to boscalid (a fungicide) and zinc (a heavy metal) within 24 h of a sublethal exposure while not observing any physical changes in outward appearance [8]. Omics data therefore have the potential to provide informative, contaminant-specific biomarkers for environmental assessments, especially if more than one level of data is used. 

While metabolomics can measure the biochemical stresses induced by individual compounds, such as fungicides, in waterways [9,10,11], there is considerable concern that when contaminants occur as a mixture different metabolomic pathways will be affected when compared to single exposures. This could result in any chemical-specific indicators developed from single exposures becoming invalid for mixture assessments [1,12]. This concern stems from the ability of contaminant mixtures to act additively, synergistically, or antagonistically to influence metabolomic responses (or not interact at all). For example, Vandenbrouck et al. studied *Daphnia magna* exposed to two polycyclic aromatic hydrocarbons (pyrene and fluoranthene), both singly and as mixtures [13]. The results showed that the biochemical changes induced by exposure to these pollutants were not the same when the two compounds were applied singly compared to a mixture [13]. This meant that metabolites of exposure developed in the single toxicant experiments did not apply to the mixture tests and were therefore not useful environmental biomarkers. 

The Vandenbrouck et al. study used NMR and Gas Chromatography–Mass Spectrometry (GC–MS) based testing because the use of only one analytical approach has the potential to miss detecting some metabolites due to the selectivity and sensitivity of the instruments. Similarly, the present study explored the metabolomic changes in *Simplisetia aequisetis* following exposure to pollutants using a multi-platform, GC–MS and Liquid Chromatography–Mass spectrometry (LC–MS) approach. The two contaminants assessed (zinc and boscalid) were chosen because they are widely detected in estuarine sediments in Victoria, Australia. Zinc may be both natural and anthropogenic in origin, while the source of Boscalid (a fungicide) is predominantly agricultural run-off [14]. Both pollutants commonly occur in ecosystems as mixtures, but the combined effect(s) of these two compounds on the environment is not well understood [15,16,17]. A recent review by O’Brien et al. did however, highlight the necessity for research on the effects of multiple stressors on estuaries and marine ecosystems [18]. O’Brien et al. noted that the effects of single contaminant stressors are evident from decades of research and that research needs to now determine how these contaminants are affecting ecosystems in mixtures [18]. The results from this study shed new light on zinc and boscalid toxicology, adding to the limited toxicological information available for estuarine ecosystems. The work illustrates the potential of metabolomics to form connections from mixture exposure to biochemical responses of organisms in a controlled setting of the laboratory to the more dynamic, real-world environmental field assessments. 

## 2. Results and Discussion

### 2.1. Multivariate Analysis of Metabolites

Fifty-five metabolites were detected and quantified from the GC–MS, including compounds involved in amino acid metabolism, the tricarboxylic acid (TCA) cycle and the urea cycle. Permutational Multivariate Analysis of Variance Analysis (PERMANOVA), a statistical tool used for the analysis of multivariate data on the basis of Euclidean distances dissimilarity measures, was used to assess data from both GC–MS and LC–MS approaches. The PERMANOVA mirrors a standard analysis of variance (ANOVA) but it is more suitable to complex experimental designs [19]. 

Surprisingly, the PERMANOVA indicated no significant difference in metabolites across the four treatment groups when they were compared together (Table 1) although the lack of significance was marginal (*P* = 0.069). A pairwise comparison between exposure groups showed a significant biochemical difference between the zinc and mixture treatments (*P* = 0.03). Boscalid exposure, in contrast, had no significant effect (*P* = 0.14–0.30). The data were later explored further, using univariate methods.

**Figure 1:**
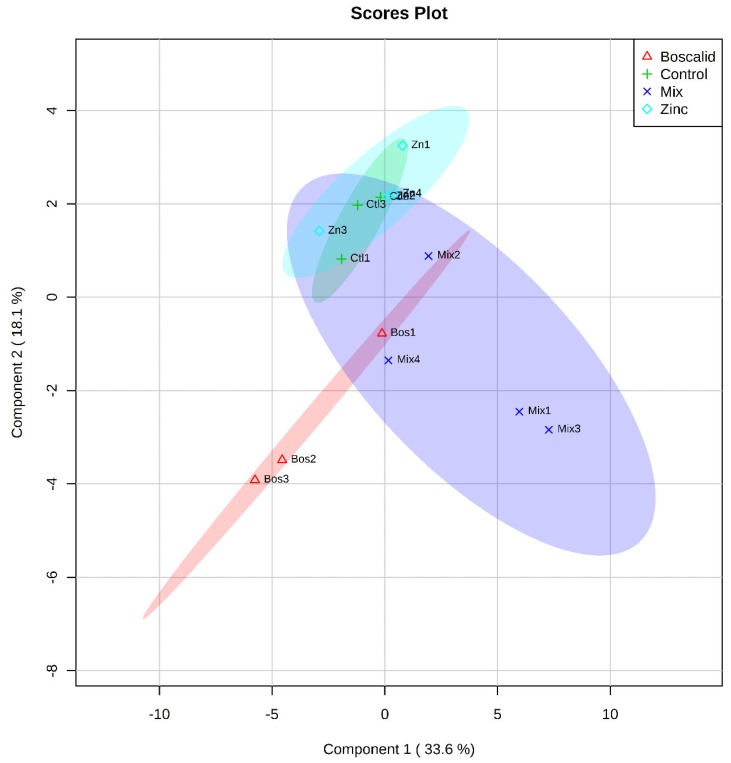
A partial least squares discriminant analysis: separation of identified metabolites from whole annelids (*S. aequisetis*) across treatment groups, following a GC–MS approach. Cross-validation data can be found in the supplementary material (Figure S1 and Table S3).

**Figure 2:**
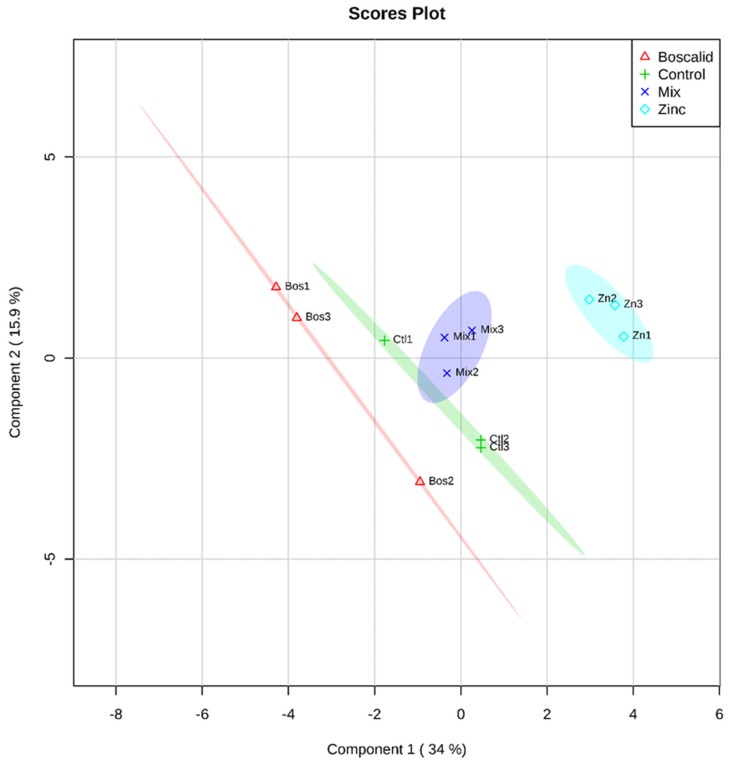
A partial least squares discriminant analysis: separation of identified metabolites from whole annelids (*S. aequisetis*) across treatment groups, following an LC–MS approach. Cross-validation data can be found in the supplementary material (Figure S2 and Table S4).

A partial least squares (PLS) analysis was also undertaken to check for small changes that might not be indicated by PERMANOVA. The PLS is a visualisation of multivariate analysis relationship between metabolites and treatments as a variable following a cross-validation (CV) procedure detailed in Szymańska et al. [20] (Figures S1 and S2, Tables S3 and S4)). The zinc and boscalid single exposure, showed a clear separation in the PLS, using both GC–MS (Figure 1) and LC–MS (Figure 2) data. The zinc and control treatments overlapped in the GC–MS data, but the mixture treatment did not show separation from the other treatments. In contrast, the PLS analysis did show clear separation between all treatments following the LC–MS approach (Figure 2), indicating that annelid metabolites had trends in abundance unique to each treatment. This also highlights the information that can be gained using metabolomics to investigate the initial effects contaminants may have on organisms. 

Forty-nine metabolites were identified using an LC–MS approach. These compounds again included those contributing to the TCA cycle, the urea cycle, and several other metabolic pathways. Metabolite systems are usually under tight regulatory control, so even a small change may induce large biological effects. The observed changes in the TCA and urea cycle could therefore be biologically relevant. The size of the effect caused by each treatment is difficult to equate to biological relevance, as the molecular functioning of *S. aequisetis* is not yet defined, but even small disruptions in pathways can lead to substantial changes in the overall health of organisms. 

The univariate ANOVA analysis identified ten metabolites (Table 2) from the LC–MS data that changed significantly. The multivariate PERMANOVA analysis identified this change to be likely caused by the treatment variable (*P* = 0.0006) (Table 1). Interestingly, a pairwise comparison of the treatments showed no significant effect between any comparisons (Table 1). 

### 2.2. Univariate Analysis of Treatment Effects on Metabolites Responses

Univariate analysis was undertaken to assess the changes in the relative abundance of individual metabolites from each treatment group. To identify metabolites that changed significantly (*p* < 0.05) following exposure, a one-way ANOVA, using treatments as a factor, was run for each metabolite (Table 2). Following the ANOVA, a Tukey’s multiple post hoc test revealed zinc and mixture treatments caused the abundances of more metabolite to be significantly affected compared to the singular boscalid treatment (Table 2). The ANOVA further highlighted 17 metabolites, from both analytical platforms, that were significantly affected by treatments. There were three metabolites that were detected by both platforms to change significantly due to toxicant exposures (*p* < 0.05). Two of these were serine and threonine, which contribute to the TCA cycle. The third was ornithine, part of the urea cycle. The reason for the differences between the metabolites identified as significant using each method is unclear but demonstrate the importance of using a multiplatform approach wherever possible [21,22].

### 2.3. GC–MS Data

**Figure 3:**
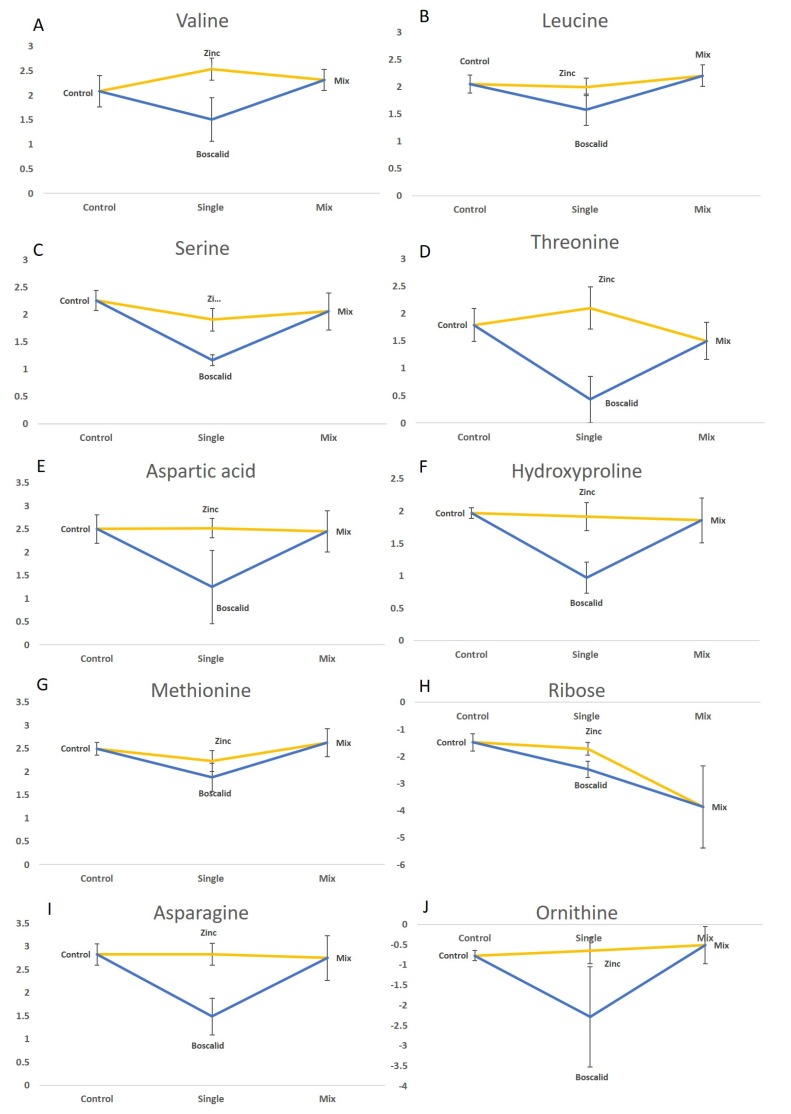
Line graphs of metabolites which significantly changed in abundance (*p* < 0.05) following whole annelid (*S. aequisetis*) single exposure to zinc, boscalid and to their mixture. (**A**) Valine; (**B**) Leucine; (**C**) Serine; (**D**) Threonine; (**E**) Aspartic acid; (**F**) Hydroxyproline; (**G**) Methionine; (**H**) Ribose; (**I**) Asparagine; and (**J**) Ornithine. Y axis shows the peak area abundance for each metabolite from the GC-MS analysis.

Four metabolites were found to have been significantly affected by all treatment comparisons, these were serine, threonine, hydroxyproline, and asparagine (Table 2). Serine and threonine are non-essential amino acids that contribute to glycine metabolism, glycolysis, and the transsulfuration pathway. Importantly, both serine and threonine contribute to the TCA cycle, the main form of energy production in most organisms. Changes in TCA cycle metabolites are a very common response to toxicant exposure or stress [23,24,25] so are not useful biomarkers for specific exposures. Serine is also involved in fatty acid metabolism and the maintenance of immune system responses [26,27]. Hydroxyproline is the result of protein degradation. The most reported source of hydroxyproline is collagen, which is the main structural protein in the extracellular space [28]. Asparagine is essential in coordinating overall protein synthesis, cellular responses to amino acid homeostasis, and metabolic availability during biological processes and disease development. For example, asparagine acts as a metabolic regulator of TCA cycle intermediates and asparagine bioavailability is essential for survival. [29]. The abundance of these metabolites after boscalid exposure was reduced compared to the control, mixture, and zinc treatments (Figure 3). There was not any statistically significant change detected in the mixture treatment of these metabolites. There also wasn’t any statistically significant effect of zinc or mixture on aspartic acid, hydroxyproline, asparagine, and ornithine abundance (Figure 3). Ribose, which is used in the synthesis of nucleic acids and metabolic intermediates for biosynthetic processes that prevent oxidative stress [30], was significantly lower in the mixture treatment; but at *p* = 0.045, this difference is unlikely to be truly significant and any potential impact this change may be having on the organism’s health is likely small.

Two metabolites (serine and methionine) increased in relative abundance following the mixture exposure compared to the single treatments. Methionine is an α-amino acid described in Deutz et al. as an important compound for protein synthesis, the formation of polyamines, and its involvement in the synthesis of many metabolites, including homocysteine [31]. Methionine is also involved in detoxification responses through the transsulfuration pathway and the production of metallothionein [10]. Both serine and methionine decreased in relative abundance when annelids were exposed to zinc as a single treatment (Figure 3). This result correlates with the responses seen in *Chironomus tepperi* after being exposed to zinc at similar concentrations in Long et al. [10]. Since the changes in serine and methionine abundance levels due to zinc exposure vary depending on whether it is given as a single exposure or as a mixture, it is unlikely that these two metabolites will be useful as field biomarkers for zinc exposure without further research [32,33,34].

The results of this study indicate that overall toxicity was reduced when compounds were applied as a mixture exposure and that zinc had a slightly stronger effect than boscalid on certain metabolites. Similar results were seen with the toxicity interactions of chlorpyrifos (insecticide) and nickel (heavy metal) in a mixture exposure to marine bivalves, using a transcriptomic approach, investigated by Dondero et al. [35]. Their results also demonstrated that the mixture treatment had unexpected responses compared to the single exposures. They also found an overall decrease in toxicity when the contaminants were presented as a mixture [35]. In both the current study and previous studies, the changes in toxicity could be due to detoxification mechanisms being primed by the first exposure and so later being able to deal with subsequent exposure to any toxicant [36,37,38].

### 2.4. LC–MS Data

**Figure 4:**
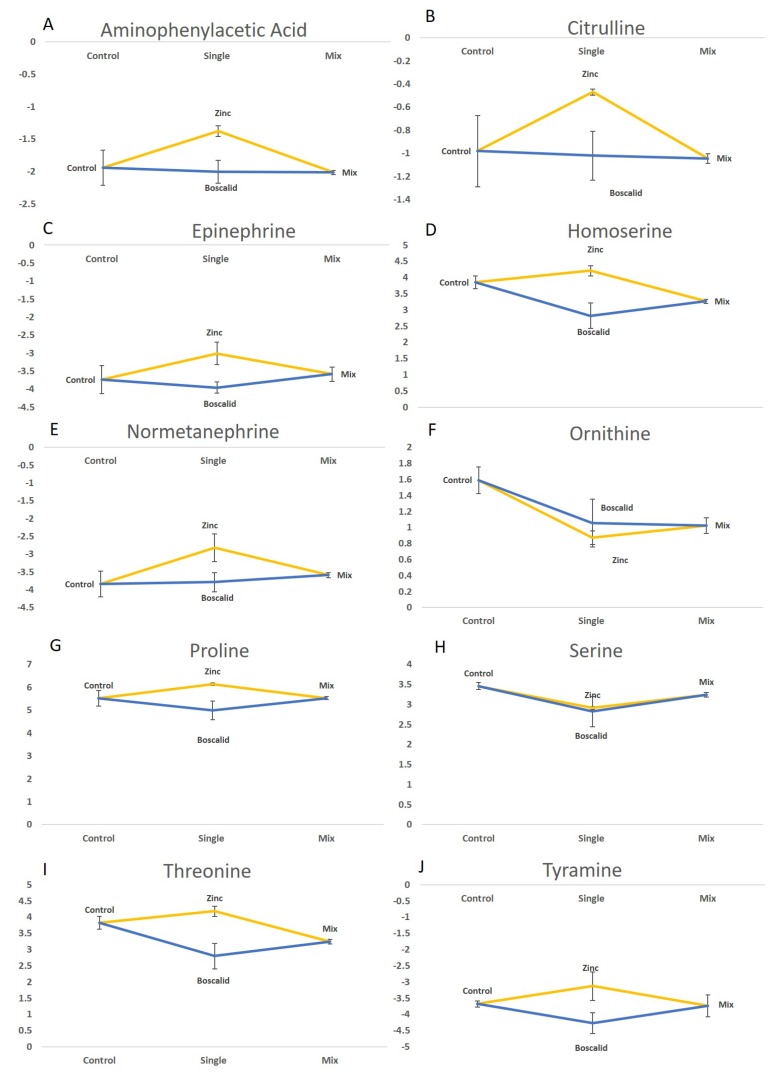
Line graphs of metabolites which significantly changed in abundance (*p* < 0.05) following whole annelid (*S. aequisetis*) single exposure to zinc, boscalid and to their mixture using LC-MS data: (**A**) Aminophenylactic acid; (**B**) Citrulline; (**C**) Epinephrine; (**D**) Homoserine; (**E**) Normetanephrine; (**F**) Ornithine; (**G**) Proline; (**H**) Serine; (**I**) Threonine; and (**J**) Tyramine. Y axis shows the peak area for each metabolite from the LC-MS analysis.

Metabolomic analysis via LC–MS had a higher sensitivity and selectivity compared to GC–MS-based screening. For example, ornithine was detected using both methods, but while LC–MS analysis identified a significant change in abundance of this compound between treatments (*p* = 0.014), GC–MS did not (*p* = 0.05) (Table 2). Ornithine is a main component of the urea cycle (also known as the ornithine cycle) which produces urea from ammonia and is essential for survival. Threonine abundance was trending higher following zinc exposure and lower following the boscalid exposure compared to controls. Interestingly, the effect of the mixture exposure on threonine was similar to the boscalid response, with decreasing levels compared to controls. Threonine, like serine, is a major metabolite in the TCA cycle. Ornithine and serine had a reduced abundance following exposure to all treatments, indicating that the urea cycle could have been disrupted by the organism’s attempting to detoxify the contaminants (Figure 4) [39].

The metabolites from LC–MS, such as proline, tyramine, normetanephrine, epinephrine, and aspartate, increased in abundance as a response to zinc and decreased following boscalid treatment (Figure 4). Interestingly, the abundance of these compounds in the mixture exposures was the same as in the controls. Citrulline levels were increased in the zinc treatments but remained the same as controls following exposure to boscalid and the mixture treatment. Citrulline is a primary metabolite in the urea cycle, which is consistent with the pathways suggested to be affected by exposure to a heavy metal from the GC–MS. The difference in citrulline abundance in the mixture treatment may indicate a faster detoxification response occurring when annelids are exposed to two contaminants compared to that of single treatment of zinc. 

### 2.5. Combined Metabolite Pathway Responses

The pathways affected in the current study are consistent with metal and fungicide exposure responses described in previous research [8,10,14]. This indicates that the biochemical responses seen may be common across species and thus have the potential to be indicators of zinc and boscalid exposure generally. When the compounds were exposed as a mixture, the changes in the metabolites tended to be similar to the controls or follow those of the stronger contaminant. For example, in the present study, levels of serine increased following exposure to a mixture and decreased in response to the zinc and boscalid exposure. Similarly, Jones et al. found that when nickel and the organophosphorus insecticide, chlorpyrifos (Chlp) were mixed, the effect on the metabolic profiles resembled those due to Chlp and the responses to nickel were not detected [40]. Interestingly, Jones et al. determined that the biochemical changes that occurred in the mixture were also different from the single treatment metabolic response [40]. Comparable results were seen by Baylay et al., using mixtures and single exposures of thiacloprid and imidacloprid [12]. 

When an organism is under stress, the primary function of many compounds is altered [41]. The alteration of metabolite primary functions could explain changes in metabolite relative abundance seen in this study. Alternatively, the differences in metabolite abundance could be due to the addition of boscalid, following a week of zinc exposure. In the latter case, the allocation of resources by annelids to respond to zinc exposure was already underway when the fungicide was added; therefore, the response to boscalid was not as large as might be expected [36,37,38,39,40,41,42,43,44,45,46,47,48].

It is challenging to determine how the different responses of these metabolites may contribute to the overall functioning of the organism. The current study analyzed steady-state metabolomics, so it is therefore difficult to extrapolate the trending direction of the measured metabolites with absolute confidence, whereas metabolomic flux studies could show metabolite changes that were lacking in the mixture exposures. There are, however, many similarities between the present study and the metabolite responses of invertebrates’ exposure to pollution documented in the literature, such as the responses detected affecting the TCA, energy metabolism, and transsulfuration pathways [10,42]. The detected shifts in relative abundance of metabolites can be attributed to shifts in key metabolic processes, such as energy synthesis and other signaling molecules. These molecules are involved in the regulation of growth, metabolism, and stress responses. Disruption of normal cell function caused by zinc and boscalid creates an increase in detoxification and energy production to continue regulation via the TCA cycle and other metabolic processes [25,31]. 

## 3. Conclusions 

This research has demonstrated how metabolomics can aid in understanding the interactions occurring between pollutants and organisms in estuarine ecosystems. Metabolomic pathways were shown to respond following exposures to single contaminants, and a mixture treatment and can, therefore, be applied as a discovery tool for molecular markers of exposure. Several key metabolomic pathway interactions were identified that might be considered when looking at mixtures, but these were not specific to the contaminants used. Changes in the TCA cycle, intermediates of the urea cycle, and the glycine, serine, and threonine cycle, are common responses to external stress seen in many studies [38,43]. An assessment framework considering multiple stressors is critical for our understanding of ecosystem responses within a stressed environment, and to inform appropriate environmental management strategies [44]. Mixture toxicity is an ongoing global issue that requires further investigation of molecular responses of organisms to complex chemical mixtures if we are to effectively manage anthropogenic contamination occurring in environments such as urban estuaries. 

This work has highlighted the need to be able to unravel the complex chemical and biological interactions that occur in the environment in more depth. Future research should investigate long-term exposure to chemical mixtures and include the effects of secondary doses of toxicants. Incorporating other individual fitness measures, such as reproduction and weight changes, as well as population-level metrics [45], would provide a greater understanding on the overall effects of contaminants at an ecological level. 

Metabolomics can provide sensitive detection of initial early warning responses for ecotoxicology experiments or field assessments. The current research highlights the importance of using a multiplatform approach to enable the detection of a broader range of sensitive responses indicating the overall health and functioning of *Simplisetia aequisetis* [10,20,21,27]. Incorporating, steady-state metabolomics with metabolic flux studies using ^13^C/^15^N-labelled substrates to understand the mechanisms and map the metabolic changes occurring within an organism in the future would provide further information on the modes of action of specific chemicals [46]. Metabolomic technologies can offer an understanding of the biochemical basis of toxicity and the mode of action chemicals take within an organism [7,10,47,48]. The application of initial warning biomarkers for mixture exposures can be used in conjunction with other biomonitoring tools to identify sources, toxicity, and uptake of contaminants. Early indicators could enable prioritisation of major contamination issues to support targeted conservation efforts for estuarine habitats around the world.

## 4. Materials and Methods 

### 4.1. Test Species

*Simplisetia aequisetis*, an estuarine annelid, was collected from Little River, Victoria, Australia (about 50 km south-west of the Melbourne central business district). *Simplisetia aequisetis* were chosen for this study because they are abundant, easy to collect, an important food resource in estuarine food webs (for example, for fish species and birds), and they spend their life cycle in the top layers of the sediment, where exposure to contaminants is most prevalent. All annelids collected from the environment were transported and maintained within the University of Melbourne marine laboratory, where they were kept in a controlled environment with recirculating ambient seawater and Little River sediment with the following experimental conditions: temperature (18 ± 1 °C), salinity (34.2 ppt), and a 16:8 h light:dark photoperiod. All annelids were left to acclimatise for a minimum of seven days and fed once a week with ground tropical fish flakes (Tetramin^®^, Tetra Werke, Melle, Germany) before experiments began. 

### 4.2. Mixture Exposure

Annelids were exposed to 12.5 mg/L of ZnCl for a week and were then dosed with 75 mg/L of boscalid (applied as the commercial product Filan^®^, Nufarm, Melbourne, Australia, 500 g boscalid per kg of product) for a total of two weeks. Filan^®^ (Nufarm, Melbourne, Australia) was used instead of pure compound as it is water soluble so reduces the need to use external solvents that might have impacted the toxicology experiments. Eighteen grams of sieved (250 µm) frozen and thawed sediment (to ensure no live organisms were indirectly included in the test sediment) collected from the Little River site was used as substratum for each beaker, and 300 mL of each test solution was prepared with marine water and zinc stock solutions. On the day of the experiment, *S. aequisetis* were counted, by sieving the sediment with a 250 µm mesh, sorted by size, and placed into seawater to remove any debris. Three randomly selected annelids (6 cm ± 1 cm in length) were added to each 600 mL beaker and fed 0.5 mg of ground Tetramin^®^ (Tetra Werke, Melle, Germany) per beaker at the beginning of the experiment. Plastic cling-wrap was placed over the top of the beaker to prevent condensation and changes in salt concentration. Gentle aeration using glass pipettes was provided over the course of the experiment to maintain dissolved oxygen levels between 70% and 100%.

The beakers were randomly positioned in a temperature-controlled room at 18 ± 1 °C (reflecting the environmental conditions from where the individuals were collected), with a photoperiod of 16:8 h, light:dark. A zinc-only exposure of 12.5 mg/L ran for one week (*n* = 10 replicate beakers) with seawater-only controls (*n* = 10 replicate beakers). At the start of the second week, to simulate a ‘runoff event’ [49], 75 mg/L of boscalid (Filan^®^) was added half a centimeter below the surface of five of the zinc treatments and five of the controls. Exposure concentrations were based on 10% of the acute toxicity concentration (LC_50_) in sediment to *S. aequisetis* (Zinc chloride LC50 = 125 mg/L and boscalid LC_50_ = 750 mg/L) (Table S1) [8]. The experimental concentrations were higher than the levels of zinc and boscalid detected in wetlands and estuaries around Victoria, Australia, which range between 0.111 and 3.41 mg/L for zinc [10] and between 0.022 and 3.3 mg/L for boscalid [14], to increase the likelihood of a metabolic response. 

The experiment ran for 168 h in a temperature-controlled room. At the end of the experiment, annelids were collected from the separate treatments of zinc (*n* = 5), boscalid (*n* = 5), the mixture (*n* = 5), and the control (*n* = 5). Water quality parameters (dissolved oxygen, temperature, electrical conductivity, and ammonia levels) were measured at the beginning and end of experiment to ensure that no substantial changes in conditions had occurred (Table S2). 

### 4.3. Sample Preparation 

The annelids were gently rinsed in distilled water to remove debris and blotted dry on tissue paper to remove excess water that could impact subsequent metabolomic analyses. Organisms were pooled per replicate and placed in pre-weighed 2 mL microcentrifuge vials and frozen on dry ice within 30 s of removal from test beakers. Each sample contained pooled whole annelids, which were ground to a fine powder under liquid nitrogen using a mortar and pestle. A total of 30 mg of the homogenised sample was placed into pre-weighed and precooled 2 mL lysing tubes containing 1.4 mm ceramic lysing beads (Bertin Technologies, Aix En Provence, France) and returned to −80 °C. The remaining tissue was re-weighed and stored at −80 °C.

### 4.4. Metabolite Extraction 

Metabolites were extracted using a modified Bligh–Dyer method, as described in Sinclair et al. [8]. Briefly, annelid tissue samples were first lysed at 6800 rpm (3 × 45 s), using a Precellys bead-mill attached to a Cryolys cooling unit (Bertin Technologies, Aix En Provence, France) that had been pre-chilled with liquid nitrogen. The samples then had 600 µL aliquot of methanol:chloroform (9:1 ratio) solution containing an internal standard (140 µM ^13^C5 -^15^N-Valine and 14 µM ^13^C6-Sorbitol) added to them) The tubes were then thoroughly mixed and centrifuged at 14,000 rpm at 0 °C for 15 min to generate upper (aqueous) and lower (organic) phases. A 400 µL sample of the upper phase was collected into a clean microcentrifuge tube. A 100 µL aliquot from each sample was taken for GC–MS analysis and a 10 µL aliquot was taken for LC–MS analysis. 

### 4.5. Gas Chromatography-–Mass Spectrometry (GC–MS) and Liquid Chromatography–Mass Spectrometry (LC–MS)—Amine Compounds

The metabolites identified from both analytical approaches, GC–MS and LC–MS, are listed in supplementary material (Table S5).

The extracts for both GC–MS and LC–MS analysis followed the procedure detailed in Sinclair et al. [8]. Briefly, a 25 µL aliquot was taken from all sample extracts and pooled to create a biological quality control (PBQC), which was then aliquoted into five replicates. All samples were dried and then derivatised online, using a Gerstel MPS2 XL autosampler robot (Mülheim an der Ruhr, Germany) for the GC–MS analysis, as described in Sinclair et al. [8]. GC–MS analyses was conducted using an Agilent (Agilent, Santa Clara, CA, USA) 7890A gas chromatograph coupled to an Agilent 5975 C mass spectrometer (Agilent, Santa Clara, CA, USA) with a Gerstel Autosampler (MPS 2 XL) [10]. A semi-targeted profiling analysis as described in Overgaard et al. was performed with Agilent MassHunter Quantitative Analysis software (B.07.0) to produce an integrated area matrix [50]. Metabolites were identified using an ‘in house’ standard list (Table S6) and abundance was used for statistical analysis. 

LC–MS amine analysis followed the method of Boughton et al. [51], which is also described in Sinclair et al. [8]. Briefly, the supernatant was diluted with methanol and buffered by the addition of borate buffer [10], containing 13C3 -L-alanine (Sigma–Aldrich, St. Louis, MO, USA) as the internal standard. AQC reagent (6-aminoquinolyl-N-hydroxysuccinimidyl carbamate, 10 mM in 100% acetonitrile) was added to each sample and then incubated for 10 min at 950 rpm at 55 °C in a thermomixer. For A QC derivatisation, samples were centrifuged at 14,000 rpm at 0 °C [51]. The LC–MS approach was undertaken on a Shimadzu 8060 series Triple Quad LC–MS system (Shimadzu Scientific Instruments (SSI) Kyoto, Kyoto Prefecture, Japan). The column used was a 120 SB-C18 Poroshell (2.1 × 100 mm, 2.7 micron). The method was set up using MRMs (multiple reaction monitoring) for specific metabolites and the data were processed using Shimadzu Lab Solutions Version X (SSI). Relative abundances were calculated by manual curation of all MRMs detected, and IDs were confirmed using a standard mix (Table S7) of amine-containing metabolites analyzed alongside the samples. 

### 4.6. Statistics

Statistical analysis followed the procedure described in Sinclair et al. [8]. GC–MS and LC–MS metabolite values were normalised to the median value of each sample and then transformed (log(natural)) to reduce variance between metabolite abundance. The data from each approach were analyzed separately. Multivariate data were analyzed using a factorial permutational multivariate analysis of variance (PERMANOVA) based on a Euclidean distance resemblance matrix, using PRIMER software (Version 7, Massey University, Auckland, New Zealand) [52]. Principle component analysis (PCA) was initially considered for visualisation of metabolomic separation, however, it was not used due to the sensitive biological variables affecting metabolites between each individual annelid varying more than the treatments. This created difficulties detecting differences across treatments. The use of Partial least squares (PLS) and Partial least squares (PLS) discriminate analysis (PLS–DA) (with appropriate validation) is a common method for determining separation in metabolomic data sets due to treatments when biological natural variation outweighs treatment variation [20]. Partial least squares (PLS) analysis was carried out using online Metaboanalyst software (Version 4.0, Xia Lab, McGill University, Anne de Bellevue, Quebec, Canada) [53], using both GC–MS and LC–MS data sets to visualise overall separation of treatments [54]. The PLS–-DA method was run and cross-validated according to Szymańska et al. [20]. For the cross-validation (CV), Q2 is an estimate of the predictive ability of the model. In each CV, the predicted data are compared with the original data, and the sum of squared errors is calculated. For more details on the CV method, please refer to Szymańska et al. [20]. Multivariate *p*-values are listed using capital ‘*P*’ and univariate as a lower case ‘*p*’.

Only metabolites that could be annotated as level 1 identifications (identified compounds by chemical reference standards), as prescribed by Sumner et al., were used for statistical analysis [55]. Individual metabolites were also analyzed using one-factor analysis of variance with treatment as the main effect.

## Figures and Tables

**Table 1:** PERMANOVA, multivariate analysis for metabolite changes to treatment as main effect. Pairwise comparison analysis for each treatment.

Source of Variation	GC–MS Metabolites			LC–MS Metabolites		
	*Df*	MS	P	*df*	MS	P
Treatment	3	72.14	0.0691	3	5.69 × 10^15^	0.0006 ^1^
Residual	10	34.973		8	1.17 × 10^15^	
	*Pairwise*	*t*	*P*		*T*	*P*
	Ctl, Bos	1.0406	0.3024		1.5462	0.2041
	Mix, Bos	1.5246	0.2004		2.4663	0.0999
	Zn, Bos	1.3158	0.1404		2.1502	0.0994
	Ctl, Mix	1.5209	0.1722		1.8296	0.1028
	Ctl, Zn	0.7768	0.7968		1.7654	0.1960
	Zn, Mix	1.6489	0.0301 ^1^		4.2502	0.0975

^1^ Values that had significant differences between treatments (*P* < 0.05). Ninety-five percent family-wise confidence level. Permutational Multivariate Analysis of Variance Analysis (PERMANOVA).

**Table 2:** One-way analysis of variance for individual metabolites, measured following exposure to treatments (zinc, boscalid, and mix).

Metabolite		MS Residual	Treatment	Comparison					
*GC–MS*	*df*	*10*	*3*	*Ctl–Bos*	*Mix–Bos*	*Zn–Bos*	*Mix–Ctl*	*Zn–Ctl*	*Zn–Mix*
Valine		0.128	0.022 ^2^	0.259	0.061	0.017 ^2^	0.843	0.409	0.821
Leucine		0.062	0.043 ^2^	0.161	0.034 ^2^	0.103	0.837	0.999	0.873
Serine		0.076	0.003 ^2^	0.003	0.008 ^2^	0.023 ^2^	0.780	0.387	0.862
Threonine		0.187	0.003 ^2^	0.014	0.037 ^2^	0.002 ^2^	0.816	0.780	0.260
Aspartic acid		0.311	0.044 ^2^	0.081	0.074	0.056	0.999	1.000	0.997
Hydroxyproline		0.086	0.005 ^2^	0.009 ^2^	0.012 ^2^	0.008 ^2^	0.957	0.995	0.992
Methionine		0.089	0.040 ^2^	0.115	0.035 ^2^	0.452	0.938	0.660	0.296
Ribose		1.006	0.034 ^2^	0.629	0.317	0.764	0.045 ^2^	0.988	0.052
Asparagine		0.178	0.006 ^2^	0.013^2^	0.013 ^2^	0.009 ^2^	0.995	1.000	0.994
Ornithine		0.587	0.050 ^2^	0.136	0.053	0.076	0.076	0.997	0.994
*LC–MS*	*df*	*8*	*3*						
Aminophenylacetic acid		0.043 ^2^	0.014 ^2^	0.983	1.000	0.024 ^2^	0.973	0.039 ^2^	0.022 ^2^
Citrulline		0.053	0.046 ^2^	0.997	0.999	0.074	0.985	0.099	0.061
Epinephrine		0.116	0.046 ^2^	0.859	0.572	0.038 ^2^	0.460	0.114	0.240
Homoserine		0.081	0.002 ^2^	0.009 ^2^	0.290	0.002 ^2^	0.132	0.468	0.016 ^2^
Normetanephrine		0.135	0.032 ^2^	0.997	0.910	0.050 ^2^	0.833	0.038 ^2^	0.125
Ornithine		0.048	0.014 ^2^	0.060	1.000	0.608	0.063	0.011 ^2^	0.589
Proline		0.106	0.018 ^2^	0.257	0.260	0.011 ^2^	1.000	0.180	0.177
Serine		0.059	0.042 ^2^	0.050 ^2^	0.231	0.968	0.701	0.094	0.404
Threonine		0.083	0.002 ^2^	0.010 ^2^	0.300	0.002 ^2^	0.138	0.473	0.017 ^2^
Tyramine		0.156	0.047 ^2^	0.321	0.401	0.031^2^	0.998	0.386	0.308

^2^ Values that had significant differences of metabolite abundance between treatments (*P* < 0.05). Ninety-five percent family-wise confidence level.

## Supplementary Materials

The following are available online at https://www.mdpi.com/2218-1989/9/10/229/s1, including a description of LC_50_ experiments, Table S1: Experiment and environment concentrations, Table S2: Water Quality Parameters, Figure S1: Cross Validation results for PLS-DA on GC-MS data, Table S3: Cross Validation table for GC-MS data, Figure S2: Cross-validation results for PLS–DA on LC–MS data; Table S4: Cross Validation table for LC-MS data, Table S5: List of 49 metabolites detected from LC–MS, 55 metabolites from GC–MS, and total list of metabolites detected and measured in the current study; and Table S6: Polar metabolite reference library, Table S7: Amine metabolite identification reference library.
