# Supplementary material for: Metabolite Changes in an Estuarine Annelid Following Sublethal Exposure to a Mixture of Zinc and Boscalid
Source: Metabolites. 2019 Oct 15;9(10):229. doi: 10.3390/metabo9100229 (PMC6835977; doi:10.3390/metabo9100229)
Supplement: Supplementary file 1 [file metabolites-09-00229-s001.pdf]

Preliminary Zinc and boscalid sub lethal concentrations

Zinc and boscalid sub lethal concentrations were determined by running preliminary 72-hour LC50 exposures. A range of concentrations, resulting in 100% survival to 100% mortality was run for both chemicals in water only exposures. Filtered seawater was used as a control and to dilute stock concentrations of zinc and boscalid. Six treatments with three replicates were run for both chemicals including a control (seawater only). The zinc chloride ($ZnCl_2$) exposure concentrations were: 62mg/L; 125mg/L; 250mg/L; 500mg/L and 1000mg/L. The commercial fungicide Filan® has 500g/kg active ingredient of boscalid, concentrations of 100mg/L; 250mg/L; 500mg/L; 750mg/L and 1000mg/L. Acid-rinsed 600ml glass beakers were randomly placed in a temperature-controlled incubator at 20°C (+/- 1°C) with added aeration and a one ply sheet of ethanol rinsed toilet paper in each beaker as substratum. No food was added; the average pH of the water was 7.5 across treatments. The dissolved oxygen remained between 70-100%. Zinc chloride LC_{50} = 125mg/L and boscalid LC_{50} = 750mg/L.

Table S1. –Concentrations of boscalid and zinc at 0 h and one week. Measured concentrations of boscalid and zinc detected in estuaries in Victoria, Australia.

	Control	Exposure at 0 hr	Exposure at 1 week	Environmental dose
Boscalid	0	N/A	75 mg/L	3.3 mg/L (Vu et al. 2016)
Zinc	0.026 – 0.034 mg/L	12.5 mg/L		3.4 mg/L (Long et al. 2015)

Table S2. Water Quality Parameters.

Date	Sample	Dissolved oxygen (%)	pH	Conductivity (µS/cm)	Ammonia
15/05/17	Control	98.36	8.44	59464.4	0.25
15/05/17	Zinc	99.10	8.31	59379.1	0.25
15/05/17	Boscalid	99.18	8.28	59656.8	0.25
15/05/17	Mixture	94.81	8.25	59386.0	0.25
30/05/17	Control	101.90	8.16	59332.4	0.5
30/05/17	Zinc	102.47	8.11	59536.7	0.5
30/05/17	Boscalid	92.17	8.15	59489.3	0.5
30/05/17	Mixture	101.57	8.14	59593.2	0.5

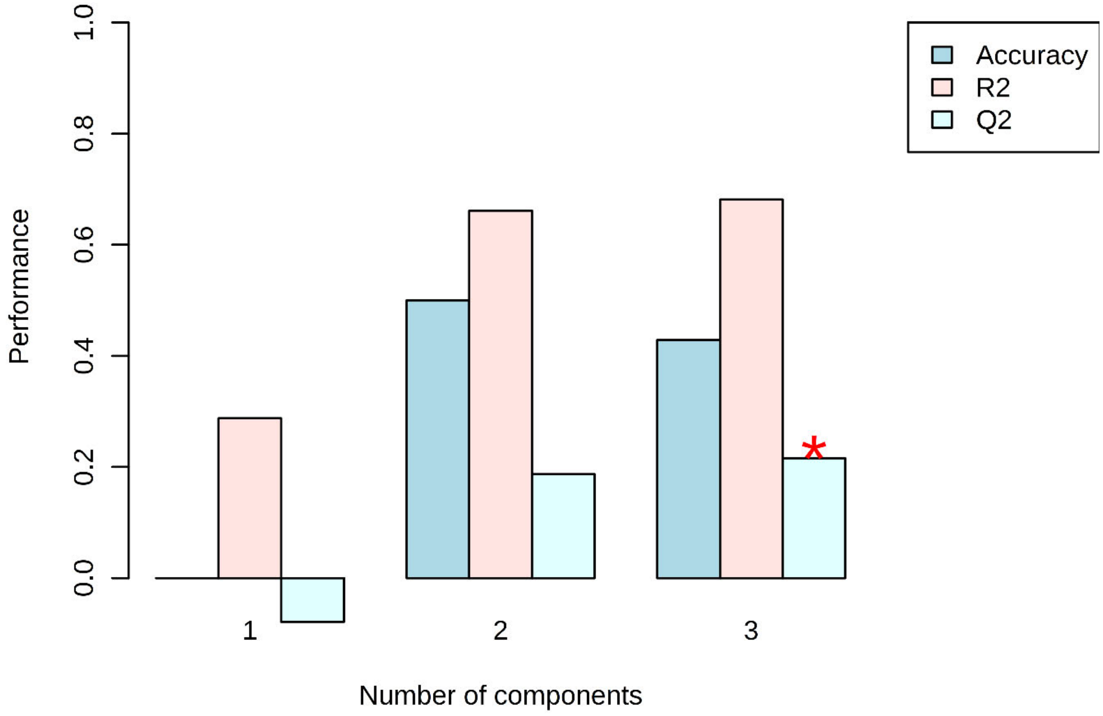

.

Figure S1. - Cross - validation graph of Partial Lease Squares from GC-MS metabolite data matrix according to Szymańska et al. [18].

Table S3. - Cross - validation figures for Partial Lease Squares from GC-MS metabolite data matrix according to Szymańska et al. [18].

PLS-DA cross validation details:

Measure	1 comps	2 comps	3 comps
Accuracy	0.0	0.5	0.42857
R2	0.28765	0.66148	0.68126
Q2	-0.079095	0.187	0.21543

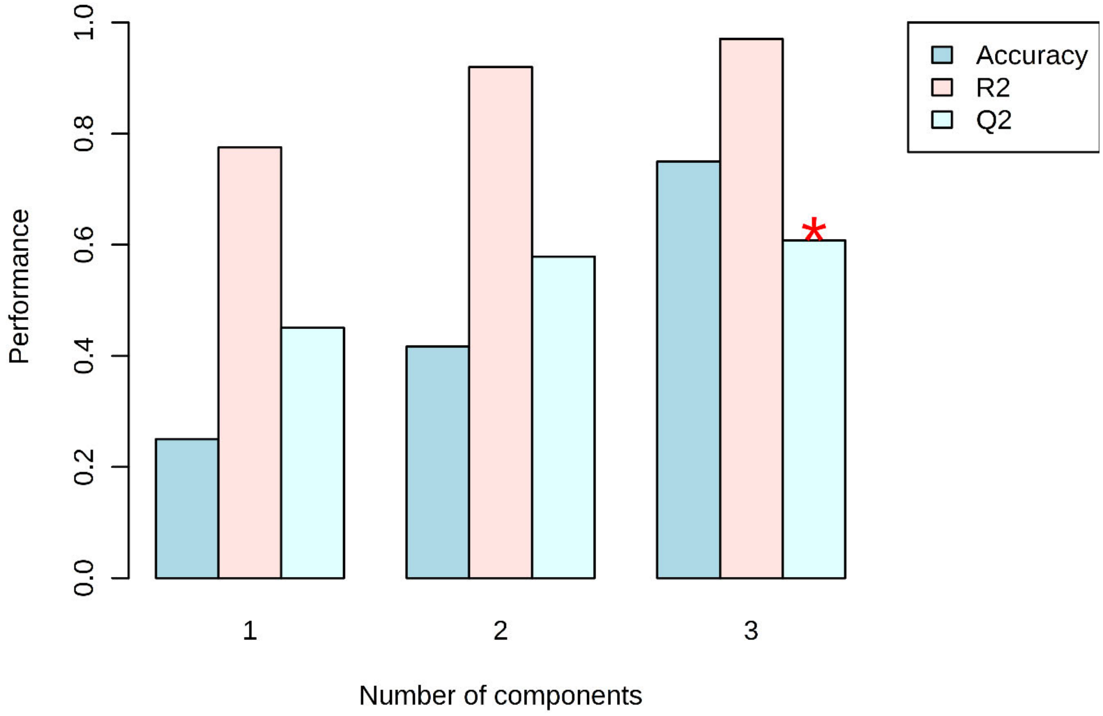

Figure S2. – Cross - Validation of Partial Least Squares from LC-MS metabolite data matrix according to Szymańska et al. [18].

Table S4. - Cross - validation figures for Partial Least Squares from LC-MS metabolite data matrix according to Szymańska et al. [18].

PLS-DA cross validation details:

Measure	1 comps	2 comps	3 comps
Accuracy	0.25	0.41667	0.75
R2	0.77563	0.91989	0.97075
Q2	0.45027	0.57855	0.60784

Table S5. - LC-MS, GC-MS and total detected and identified measured metabolites from whole worms following exposure to treatments.

LC-MS (49)	GC-MS (55)	Total (75)
Hydroxyproline	Lactic.Aci+B2:B55d	Adenine
Alanine	Oxalic.acid	Alanine
Amino.2.propanol	L.Valine	Amino Propanol
Aminophenylacetic.acid	Urea	Aminophenylacetic Acid
Ammonia	L.Serine.Pk1	Ammonia
Arginine	Benzoic.Acid	Arginine
Asparagine	L.Leucine	Asparagine
Aspartate	L.Isoleucine	Aspartate
Cadaverine.bis.AQC..M.2.	L.Proline.Pk1	Aspartic Acid
Citrulline	Glycine.Pk2	Benzoic Acid
Cystamine	Norleucine	Cadaverine
Cystathionine..M.2AQC..2	L.Threonine.Pk1	Citrulline
Dihydroxyphenylalanine	Fumaric.acid	Cystamine
Epinephrine	L.Serine	Cystathionine
Ethylamine	L.Threonine	Cystine
GABA	DL.Lactic.acid	Dihydroxyphenylalanine
g.Gly.Cys..M.Amq.h.	beta.Alanine	Epinephrine
Glucosamine	DL.Homoserine	Ethylamine
Glutamate	meso.Erythritol	Fructose
Glutamine	L.Malic.acid	Fumaric Acid
Glutathione..M.AQC..1	L.Aspartic.acid	GABA
Glycine	Hydroxyproline	Glucosamine
Homoserine	L.Methionine	Glucose
Indole.3.butyric.acid	GABA	Glutamate
Isoleucine	Pyroglutamic.acid	Glutamine
Kynurenine	D.Ribose	Glutathione
Lanthionine.2.	L.Asparagine	Glycerol 2 Phosphate
Leucine	Glycerol.2.phosphate	Glycine
Lysine.1.	Putrescine	Hexadecanoic Acid
Lysine.2.	D.3.Phosphoglyceric.acid	Homoserine
Methionine	L.Ornithine	Hydroxyproline
Normetanephrine	Tetradecanoic.acid	Indole 3 butyric Acid
Octopamine	L.Lysine.Pk2	Isoleucine
Ornithine.1.	D.Fructose.Pk2	Kynurenine
Ornithine.2.	D.Glucose.Pk1	Lactic Acid
PABA	Adenine	Lanthionine
Phenyethylamine	L.Tyrosine.Pk1	Leucine
Phenylalanine	D.Talose.Pk2	Lysine
Proline	D.Glucose.Pk2	Malic Acid
Putrescine.bis.AQC..M.2.	Mannitol	Maltose
Serine	Sorbitol	Mannitol
Spermidine.tris.AQC..M.3.	L.Lysine.Pk3	meso Erythritol
Taurine	L.Tyrosine.Pk2	Methionine
Threonine	scyllo.Inositol	myo Inositol
Tryptamine	Hexadecanoic.acid	Norleucine
Tryptophan	myo.Inositol	Normetanephrine
Tyramine	L.Tryptophan.Pk2	Octadecanoic Acid
Tyrosine	Octadecanoic.acid	Octopamine
Valine	L.Cystine	Ornithine
	Sucrose	Oxalic Acid
	Trehalose	PABA
	D.Maltose.Pk2	Phenyethylamine
	D.Turanose.Pk1	Phenylalanine
	D.Turanose.Pk2	Phosphoglyceric Acid

		Proline
		Putrescine
		Pyroglutamic Acid
		Ribose
		scyllo Inositol
		Serine
		Sorbitol
		Spermidine
		Sucrose
		Talose
		Taurine
		Tetradecanoic Acid
		Threonine
		Trehalose
		Tryptamine
		Tryptophan
		Turanose
		Tyramine
		Tyrosine
		Urea
		Valine

Table S6. - Polar metabolites, retention time and m/z in Metabolomics Australia in-house standard mix.

ns1:CompoundName	ns1:MZ	ns1:RetentionTime
Lactic Acid	117.1	6.722
L-Alanine	116	6.95
Glycine Pk1	102	7.05
Oxalic acid	190	7.06
Malonic acid	233	7.48
13C15N-Valine	149	7.56
L-Valine	144	7.58
Urea	189	7.77
L-Serine Pk1	116	7.8
Benzoic Acid	179	7.85
L-Leucine	158	7.86
L-Isoleucine	158	8
L-Threonine Pk1	117	8.032
Maleic acid	245	8.06
L-Proline Pk1	142	8.06
Glycine Pk2	174	8.09
Succinc acid	247	8.11
Norleucine	158	8.13
Glyceric acid	292	8.17
Fumaric acid	245	8.31
L-Serine Pk2	204	8.33
L-Threonine Pk2	218	8.47
DL-Lactic acid	117	8.5
beta-Alanine	174	8.74
D-Erythrose	205	8.78
DL-Homoserine	218	8.81
L-Malic acid	233	9
meso-Erythritol	217	9.054
L-Aspartic acid	232	9.17
Proline, trans-4-hydroxyl-L-	230	9.22
L-Methionine	176	9.23
gamma-Aminobutyric acid (GABA)	174	9.25
Pyroglutamic acid	156	9.26
L-Phenylalanine Pk1	120	9.45
Phosphoenolpyruvate	369	9.55
O-Phosphoethanolomine	254	9.57
Tartaric acid	292	9.71
L-Phenylalanine Pk2	192	9.77
D-Xylose	204	9.86
D-Ribose	217	9.88
L-Asparagine	116	9.92
Taurine	326	9.96
1,6-Anhydro-Beta-D-Glucose (Levoglucosan)	204	10.08
L-Rhamnose Pk1	160	10.08
Ribitol	217	10.09
Glycerol-2-phosphate	243	10.11

Supplementary Material

L-Rhamnose Pk2	160	10.13
cis-Aconitic acid	229	10.24
Putrescine	174	10.26
N-acetyl-L-Glutamic acid	84	10.44
D-(-)-3-Phosphoglyceric acid	357	10.48
Citric acid	183	10.54
Isocitric Acid	319	10.54
L-Ornithine	142	10.57
Hypoxanthine	265	10.61
D-Fructose Pk1	103	10.73
Tetradecanoic acid	117	10.73
L-Lysine Pk2	174	10.76
D-Fructose Pk2	103	10.77
D-Talose Pk1	319	10.82
D-Erythrose-4-phosphate Pk1	357	10.82
D-Gluconic Acid-Delta-Lactone	220	10.835
D-Glucose Pk1	205	10.85
D-Erythrose-4-phosphate Pk2	357	10.87
Adenine	264	10.89
D-Erythose-4-phosphate Pk3	357	10.89
D-Talose Pk2	319	10.92
Pyridoxine	280	10.93
L-Tyrosine Pk1	179	10.93
D-Glucosamine	203	10.94
D-Glucose Pk2	205	10.95
Mannitol	319	10.99
L-Lysine Pk3	174	11
Sorbitol	319	11
13C-Sorbitol	323	11.02
L-Histidine	154	11.06
L-Tyrosine Pk2	218	11.1
D-Gluconic Acid	333	11.29
scyllo-Inositol	318	11.38
Hexadecanoic acid	313	11.52
D-Ribulose-5-phosphate Pk1	357	11.63
myo-Inositol	305	11.65
D-Ribose-5-phosphate	315	11.66
D-Ribulose-5-phosphate Pk2	357	11.69
Caffeic acid	396	11.87
Spermidine	174	12.11
L-Tryptophan Pk1	218	12.17
L-Tryptophan Pk2	202	12.2
Octadecanoic acid	341	12.25
D-Galactose-6-phosphate	387	12.32
Fructose-6-phosphate	315	12.38
L-Cystine	411	12.4
Glucose-6-phosphate Pk1	387	12.43

Supplementary Material

Glucose-6-phosphate Pk2	387	12.51
D-Myo-Inositol-1-phosphate	318	12.77
6-Phosphogluconic Acid	333	12.79
Uridine	217	12.98
Sucrose	361	13.43
D-Maltose Pk1	204	13.63
D-Maltose Pk2	361	13.74
D-Turanose Pk1	204	13.76
Trehalose	361	13.77
D-Maltose Pk3	361	13.83
D-Turanose Pk2	205	13.84
Palatinose	168	14.06
Tetracosanoic acid	425	14.15
Uridine-5-diphosphate	169	14.31
Adenosine-5-monophosphate	169	14.92
Deoxycholic acid	255	16.16
Maltotriose Pk1	204	16.3
Maltotriose Pk2	361	17.34

Table S7. - Amine metabolites, retention time and m/z in Metabolomics Australia standard mix.

Metabolite	RT	m/z
Pyridoxamine	2.087	339
Ammonia	2.887	188
Glutathione [M+AQC]+1	3.23	478
4-Hydroxyproline	3.549	302
Histidine	3.761	326
Glutamine	3.924	317
Asparagine	4.255	303
Spermine terakis-AQC [M]4+?	4.272	221
Methionine	4.538	320
Lanthionine [M+2AQC]+2	4.567	275.1
Aminophenylacetic acid	4.588	322
Taurine	4.592	296
Glucosamine	4.597	350
Arginine	4.672	345
Serine	4.727	276
Ethanolamine	4.924	232
Glycine	4.93	246
Cysteamine	4.931	248
Cystamine	4.931	247
Amino-2-propanol	4.931	246
Aspartate	5.308	304
Met Sulphoxide	5.308	336.2
Agmatine	5.443	301
Creatinine	5.478	114
Creatinine 1	5.487	114
Citrulline	5.654	346
Glutamate	5.685	318
Threonine	5.882	290
Homoserine	5.883	290
Cysteine	5.884	291
Phenyethylamine	5.885	292
Norepinephrine	5.929	340
Alanine	6.293	260
Ethylamine	6.481	216
Proline	6.897	286
Trypanothione	7.04	411
Lanthionine[M+2AQC]+1	7.099	549.1
3-Hydroxytyramine	7.174	324
Octopamine	7.177	324
2-Aminobutyric acid	7.49	274
GABA	7.49	274
Homocysteine [M+Amq+h=]	7.531	306.2
Ornithine bis-AQC [M]2+	7.584	237
Ornithine bis-AQC [M]1+	7.586	473
Cystathionine [M+2AQC]+2	7.596	282.1
5-Hydroxytryptophan	7.882	391
Lysine bis-AQC [M]2+	7.963	487
Lysine bis-AQC [M]+	7.964	244
Putrescine bis-AQC [M]2+	8.013	215
Putrescine bis-AQC [M]1+	8.022	429
Epinephrine	8.18	354
Tyrosine	8.18	352
Normetanephrine	8.182	354
Tryptophan	8.38	375
Indole-3-butyric acid	8.384	374
Valine	8.386	288

Supplementary Material

Cadaverine bis-AQC [M]2+	8.437	222
Tryptamine	8.548	331
Spermidine tris-AQC [M]3+	8.654	219
Serotonin AQC [M]1+	9.026	347
Dihydroxyphenylalanine	9.255	368
g-Gly-Cys [M+Amq+h]	9.271	421.3
Kynurenine	9.355	379
Lanthionine [M+AQC]+1	9.357	379
Isoleucine	9.384	302
Leucine	9.384	302
PABA	9.385	308
Tyramine	9.386	308
Serotonin bis-AQC [M]2+	9.444	259
Phenylalanine	9.514	336
3-Methoxytyramine	9.6	338
Cystathionine [M+2AQC]+1	9.633	563.1
Cystathionine [M+AQC]+1	9.789	393.1
